# Ultrahigh-activity immune inducer from Endophytic Fungi induces tobacco resistance to virus by SA pathway and RNA silencing

**DOI:** 10.1186/s12870-020-02386-4

**Published:** 2020-04-15

**Authors:** Chune Peng, Ailing Zhang, Qingbin Wang, Yunzhi Song, Min Zhang, Xinhua Ding, Yang Li, Quanzheng Geng, Changxiang Zhu

**Affiliations:** 1 State Key Laboratory of Crop Biology, College of Life Sciences, Shandong Agricultural University, Tai’an, Shandong 271018 P.R. China; 2 Shandong Pengbo Biotechnology Co., LTD, Tai’an, Shandong 271018 P.R. China; 3 National Engineering Laboratory for Efficient Utilization of Soil and Fertilizer Resources; National Engineering & Technology Research Center for Slow and Controlled Release Fertilizers, College of Resources and Environment, Shandong Agricultural University, Tai’an, Shandong 271018 P.R. China; 4 State Key Laboratory of Crop Biology, College of Plant Protection, Shandong Agricultural University, Tai’an, Shandong 271018 P.R. China

**Keywords:** *Potato X virus*, Salicylic acid, H_2_O_2_, RNA silencing, Antivirus

## Abstract

**Background:**

Plant viruses cause severe economic losses in agricultural production. An ultrahigh activity plant immune inducer (i.e., ZhiNengCong, ZNC) was extracted from endophytic fungi, and it could promote plant growth and enhance resistance to bacteria. However, the antiviral function has not been studied. Our study aims to evaluate the antiviral molecular mechanisms of ZNC in tobacco.

**Results:**

Here, we used *Potato X virus* (PVX), wild**-**type tobacco and *NahG* transgenic tobacco as materials to study the resistance of ZNC to virus. ZNC exhibited a high activity in enhancing resistance to viruses and showed optimal use concentration at 100–150 ng/mL. ZNC also induced reactive oxygen species accumulation, increased salicylic acid (SA) content by upregulating the expression of phenylalanine ammonia lyase (PAL) gene and activated SA signaling pathway. We generated transcriptome profiles from ZNC-treated seedlings using RNA sequencing. The first GO term in biological process was positive regulation of post-transcriptional gene silencing, and the subsequent results showed that ZNC promoted RNA silencing. ZNC-sprayed wild-type leaves showed decreased infection areas, whereas ZNC failed to induce a protective effect against PVX in *NahG* leaves.

**Conclusion:**

All results indicate that ZNC is an ultrahigh-activity immune inducer, and it could enhance tobacco resistance to PVX at low concentration by positively regulating the RNA silencing via SA pathway. The antiviral mechanism of ZNC was first revealed in this study, and this study provides a new antiviral bioagent.

## Backgroud

Viruses are biotrophic parasites and important pathogens of plants and cause a range of severe plant diseases and immense annual losses of yield. To date, chemical reagents are widely used to prevent or control plant diseases in agriculture [1]. However, the extensive use of agrochemicals has caused pernicious pesticide residues and environmental pollution, declined crop quality, and threatened human health. Innovative and ground-breaking strategies are therefore urgently required to control the plant viral pathogens and improve grain output because of the growing world population [2, 3].

Biological control with endophytic strains is a promising measure to control plant diseases and eliminate pollution at the same time [4, 5]. In most cases, endophytes can produce bioactive secondary metabolites to inhibit pathogens, such as bacteria, fungi, and insects, or improve the resistance to pathogens [3, 6, 7]. The application of endophytes in biocontrol is limited because of unclear mechanism and exceptional growth condition [8]. The biocontrol agent ZhiNengCong (ZNC), which is an extract of an endophytic fungus, has been widely used in China; it is a new, efficient, environment-friendly extract. ZNC functions as an elicitor not only protecting crops from *Pseudomonas syringae pv. tomato* (*Pst*) DC3000 but also promoting crop growth [9]. In this study, we found that ZNC induces resistance to viruses at very low concentration, and the antivirus mechanism was studied.

RNA silencing is a conserved surveillance mechanism that plays a key role in defending plants against invasive nucleic acids [10–13], such as viruses, by sequence-specific degradation of complementary mRNA transcripts (post-transcriptional gene silencing, PTGS) [14, 15]. Double-stranded RNA, which is a replication intermediate generated by viral RNA-dependent RNA polymerases (RDRPs) of plant-infecting RNA viruses, triggers RNA silencing [16]. This intermediate is then cleaved into short 21–24-nucleotide small interfering RNA (siRNA) by an RNaseIII-like enzyme called dicer-like proteins (DCL) [17–20]. Double-stranded siRNAs are incorporated into RNA-induced silencing complex (RISC) containing an argonaute (AGO) protein that has an small RNA-binding domain and an endonucleolytic activity for the cleavage of target RNAs and then follows the sequence-specific cleavage of target RNAs [21].

Salicylic acid (SA) is a key plant hormone that mediates host responses against microbial pathogens. This plant hormone induces important plant defense response against pathogens [22–26]. Previous studies have shown a possible overlap between RNA-silencing pathway and signal transduction pathways governed by SA [27–30]; for example, the NtRDRP activity has been increased in tobacco plants after SA treatment [29].

In this study, *Potato X virus* (PVX) and tobacco were used as materials to study the antiviral function of the plant immune inducer ZNC. We found that ZNC induced a serious defense response in plants, including H_2_O_2_ accumulation, SA accumulation, and RNA silencing. SA biosynthesis and signaling transduction pathways are required for the ZNC-mediated defense response. This study is the first to reveal the antiviral mechanism of ZNC. The results will provide a new antiviral bioreagent and help increase the application of the plant immune inducer ZNC crop biological control in the future.

## Results

### ZNC protected plants against virus infection

**Fig. 1:**
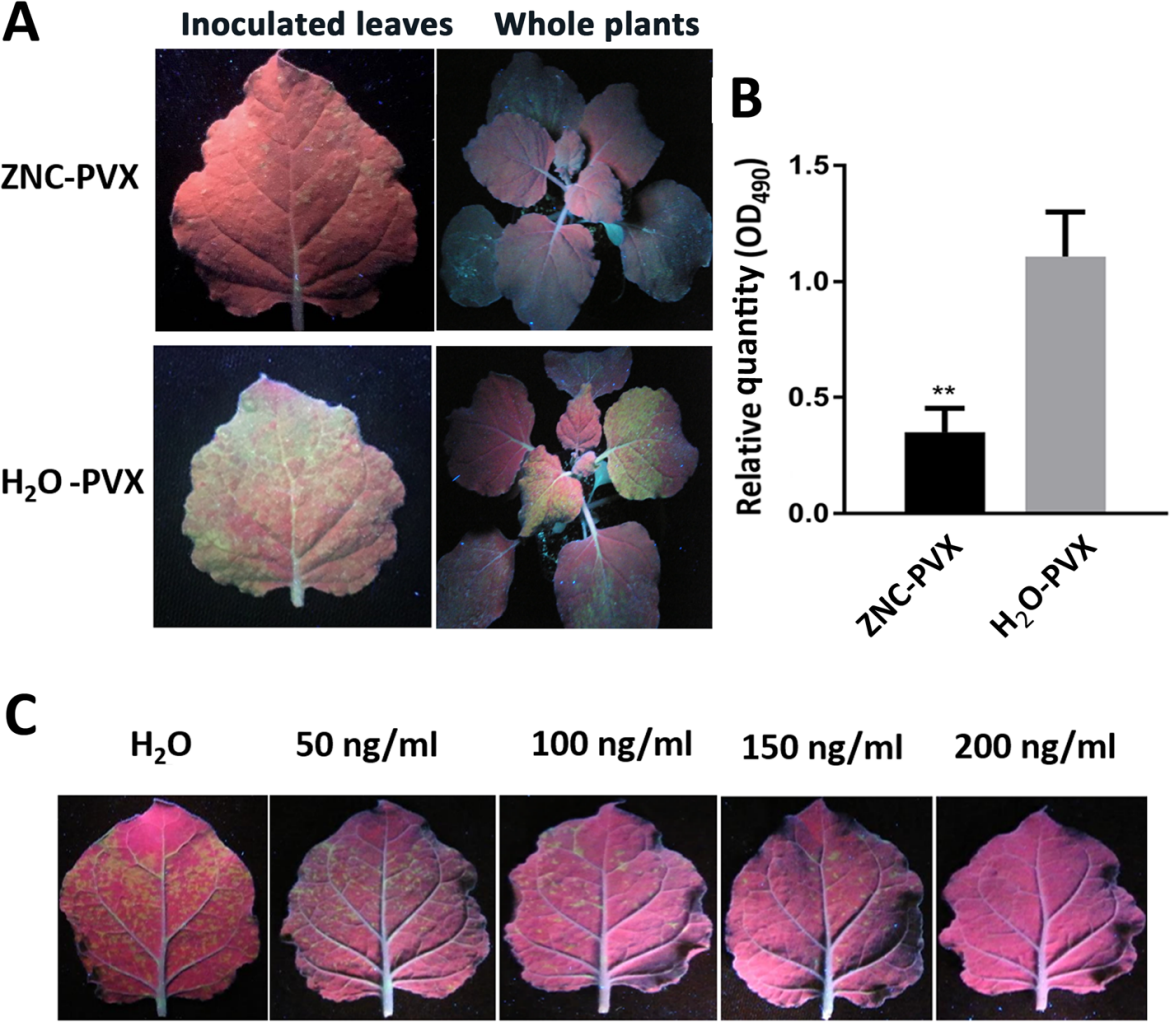
ZNC treatment enhanced plant resistance against PVX. Wild-type *N. benthamiana* was inoculated with PVX (GFP and YFP tag) after ZNC (100 ng/mL) treatment for 2 h, the phenotype (**a**) was obtained under long-wave ultraviolet lamp, and PVX relative expression quantity (**b**) was calculated using ELISA method at 5 dpi. The emerging areas were measured using a gradient concentration (0, 50, 100, 150, and 200 ng/mL) ZNC (**c**). Error bars show the mean ± SD of three replicates (at least 20 plants per replicate). ** indicates extremely significant differences determined using the Student’s t-test (*p* < 0.01)

To examine the antiviral activity of ZNC on plant, we performed an infection assay with *N. benthamiana* and PVX, which has GFP-YFP tag (Fig. S1). The H_2_O-PVX leaves (the plant was inoculated with PVX after water treatment) showed high fluorescence intensity (Fig. 1a) and large PVX quantity (Fig. 1b). The typical symptoms of virus disease occurred 5 days post inoculation (dpi). On the contrary, the symptoms of plants treated with 100 ng/mL of ZNC displayed a significant reduction in disease severity, and the relative quantity of PVX from the ZNC -PVX group was only 31.77% compared with that from the H_2_O-PVX group (Fig. 1a and b). To further confirm the function of ZNC- induced resistance against virus, leaves was inoculated with PVX before spraying ZNC and H_2_O. The results showed that ZNC could prevent virus infection to some extent (Fig. S2), and the relative quantity of PVX in ZNC treatment leaves was lower than that in the H_2_O group (74.42%). However, the difference was insignificant in comparison with the ZNC treatment (31.77%) (Fig. 1). These results suggest that ZNC prevents virus by enhancing the plant resistance to virus but does not cure the plant. Besides, ZNC also improve plant resistance to *Tobacco mosaic virus* (TMV), but have lower resistance to TMV than that to PVX on tobacco (Fig. S3).

The optimum concentration of ZNC was determined using a gradient concentration aqueous solution of ZNC at 0, 50, 100, 150, and 200 ng/mL. The results showed that the antivirus function of ZNC was gradually enhanced with the increase in its concentration. Nonetheless, when the concentration of ZNC was 200 ng/mL, the plant presented wilting. We hence used 150 ng/mL of ZNC in the next experiment.

### ZNC promoted hydrogen peroxide accumulation

**Fig. 2:**
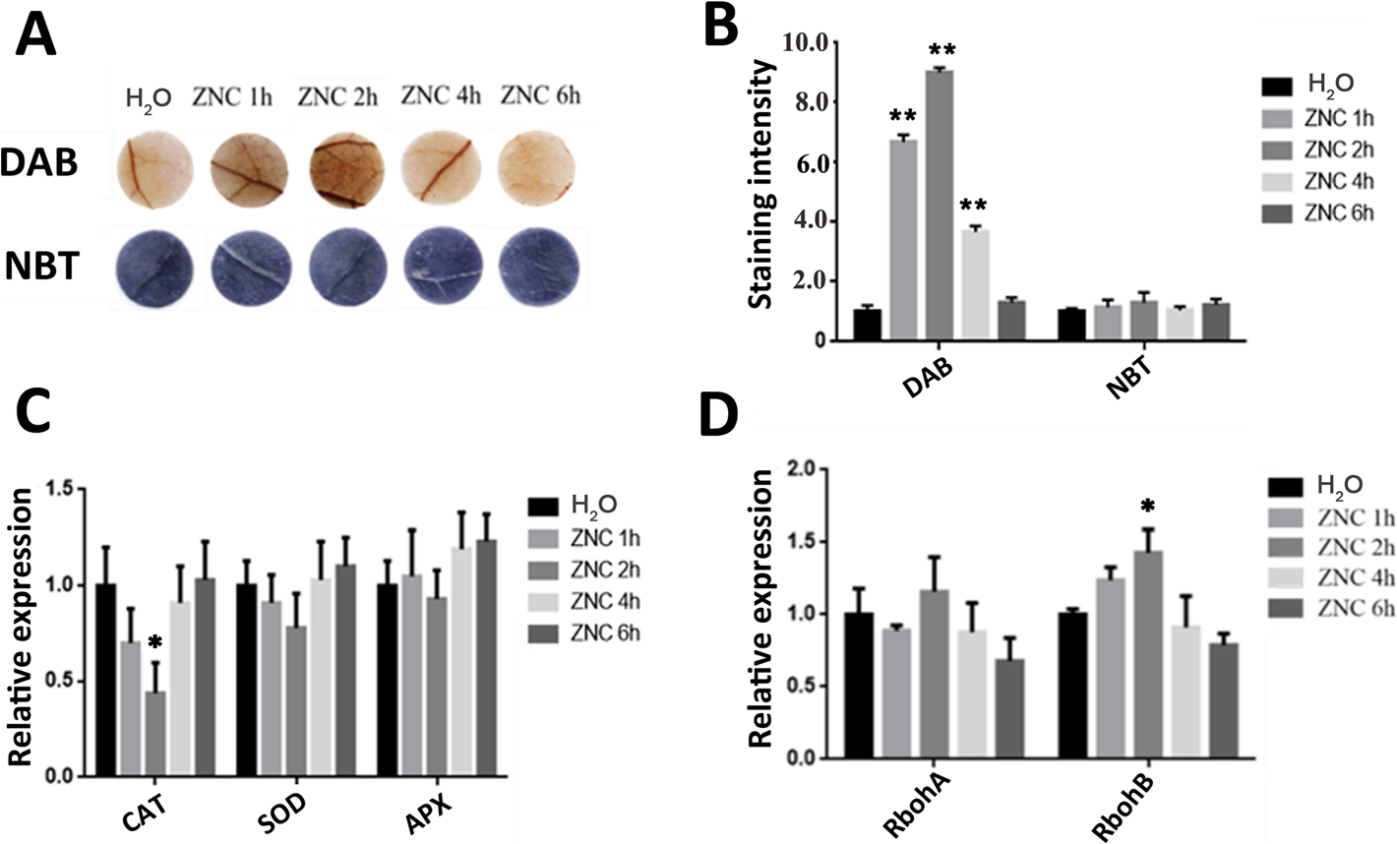
ZNC promoted hydrogen peroxide accumulation. ZNC promoted hydrogen peroxide accumulation in *N. benthamiana*. Hydrogen peroxide (up) and superoxide accumulation (down) were measured in leaves treated with 150 ng/mL of ZNC at different (0, 1, 2, 4, 6) hours post treatment (hpt) (*n* = 5). Quantification of hydrogen peroxide and superoxide levels in *N. benthamiana* treated with 150 ng/mL of ZNC at 0, 1, 2, 4, and 6 hpt. Data are shown as the mean (*n* = 5) ± SD. *CAT*, *SOD*, and *APX* were detected using qRT-PCR at various time intervals. Data are shown as the mean (*n* = 5) ± SD.qRT-PCR analysis of *RbohA* and *RbohB* expression at various time intervals. Data are shown as the mean (*n* = 5) ± SD. * indicates significant differences determined using the Student’s t-test (*p* < 0.05), ** indicates extremely significant differences determined using the Student’s t-test (*p* < 0.01).

Abiotic stresses generally enhance the production of cellular ROS and cause oxidative damage. To investigate whether ZNC regulates ROS accumulation, DAB (3,3-diaminobenzidine) and NBT staining were applied to evaluate the levels, namely, H_2_O_2_ and O^2−^, in leaves that were detached from the water-spraying group and 150 ng/mL ZNC-spraying group. Figure 2a and b, show that DAB staining was first strengthened and then weakened with the extension of time. The DAB staining was the deepest after 2 h of spraying ZNC, which indicated the accumulation of H_2_O_2_ was elevated.

The mRNA levels of several important ROS-scavenging enzymes encoding genes, namely, catalase (CAT), superoxide dismutase (SOD), ascorbate peroxidase (APX), and ROS-generating-related genes, including respiratory burst oxidase homolog genes (*RbohA* and *RbohB*), were determined by qRT-PCR analysis and monitored before and after treatment. After ZNC treatments, the expression levels of *CAT*, *SOD*, and *APX* dramatically decreased at 2 h post-treatment (hpt) compared with those in control group plants (Fig. 2c). By contrast, the expression levels of *RbohA* and *RbohB* were markedly increased (Fig. 2d). This phenomenon indicates that the accumulation of hydrogen peroxide is regulated by reducing *CAT* expression and increasing *RbohB* expression. These results suggest that ZNC may improve H_2_O_2_ production to relieve ROS toxicity but not ROS scavenging.

### ZNC promoted the SA biosynthesis and activated the SA signaling pathway

**Fig. 3:**
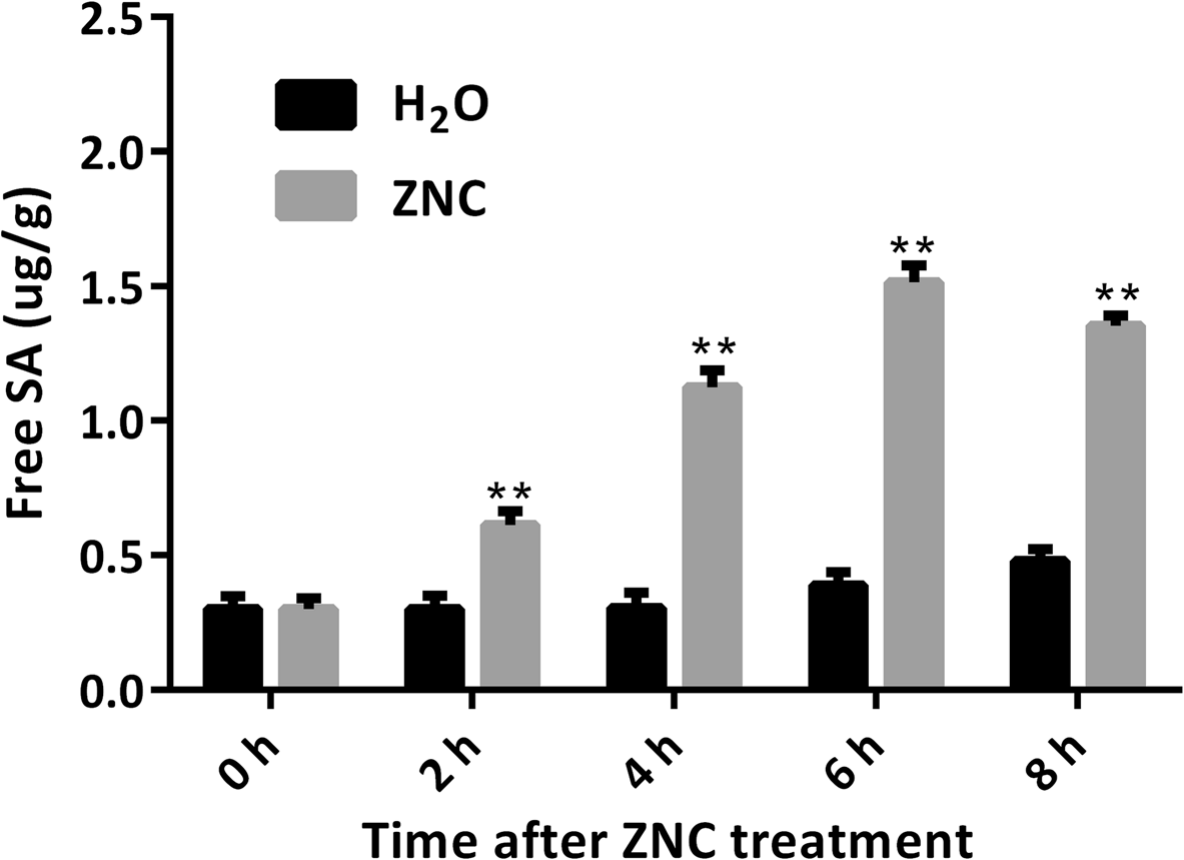
Accumulation of free SA in *Nicotiana. Benthamiana* induced by ZNC. The plants were treated with 150 ng/mL, SA was extracted at different time points. Values are the mean of three replicates ± SD (*n* = 3). ** indicates extremely significant differences determined using the Student’s t-test (*I* < 0.01).

The SA sample extraction was determined with sufficient intensity by using HPLC and gave satisfactory results. The standard addition method was used for validation because the presence of SA was detected in almost every sample (Fig. 3). The relative concentration of SA was determined by Empower software and standard SA. The changes in amount of SA induced by ZNC at different time (2, 4, 6, 8 h) in *Nicotiana*. *benthamiana* leaves were detected, and the results showed that the SA concentration was first increased and then decreased with the extension of time, and reached its concentration maximum at 6 h in Fig. 3. The results suggest that ZNC promotes SA accumulation to activate the defense response.

**Fig. 4:**
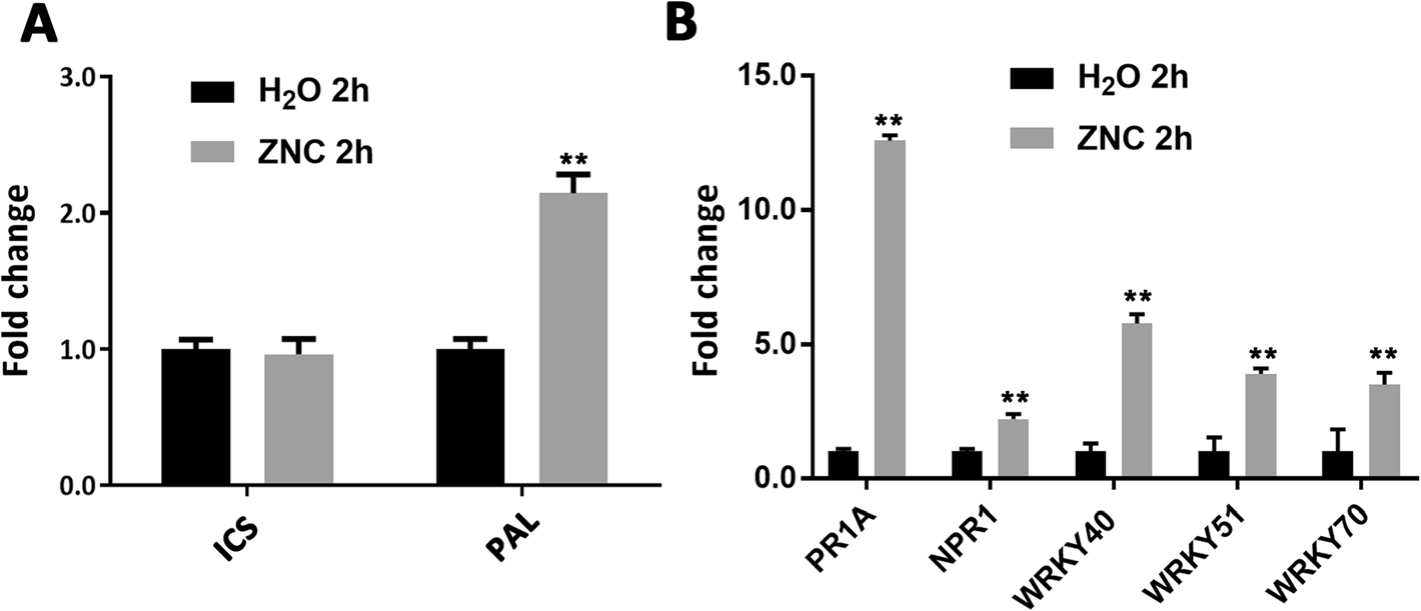
ZNC promoted SA signal transduction and SA biosynthesis. **a** SA biosynthesis-associated gene *ICS1* and *PAL* were measured at 2 hpt using qRT-PCR (means ±SD, *n* ≥ 3). **b** SA signaling-associated genes *NPR1*, *WRKY40*, *WRKY51*, and *WRKY70* were measured at 2 hpt using qRT-PCR (means ±SD, *n* ≥ 3). ** indicates extremely significant differences determined using the Student’s t-test (*p* < 0.01).

Plants synthesize SA mainly by ICS and phenylalanine ammonia lyase (PAL) pathway. Thus, we detected the *ICS* and *PAL* genes using qRT-PCR. The results showed that *PAL* gene was upregulated by ZNC, whereas *ICS* gene basically remained unchanged (Fig. 4a). These results imply that ZNC promotes SA synthesis by improving *PAL* transcription level.

Pathogenesis-related (PR) proteins play an important role in plant defense. They can improve plant disease resistance by inhibiting virus reproduction and mainly involved in plant-acquired systemic resistance. They can also be highly expressed in plants with the induction of SA [22, 31, 32]. PR-1A and nonexpressor of PR (NPR) are the key factors in the SA pathway, involved in *N. benthamiana* resistance to viruses [33] or other pathogens, such as *phytophthora* infestations [34]. WRKYs can bind the promoter region of NPR and have functions in the SA signaling pathway [35–37]. The high expression of *WRKY40* and *WRKY70* in tobacco can increase resistance to pathogens [38]; the overexpression of *WRKY70* can enhance the expression of SA-responsive *PR* genes [39]; SA is increased in *Arabidopsis* mutants by the positive regulation of *WRKY* genes, including *WRKY51* [40]*.* WRKY transcription factor genes are involved in hormone signaling pathways, such as SA pathway [41]. Furthermore, the expression patterns of several WRKY genes are significantly increased in tobacco after virus infection [42, 43]. To confirm the results, we detected the relative genes *PR-1A* (belonging to the PR1 family), *NPR1*, *WRKY40*, *WRKY51*, and *WRKY70*, by using qRT-PCR. The results showed that the expression level of the above genes was upregulated (Fig. 4b). ZNC can promote SA synthesis and activate the SA signaling pathway to enhance the defense response.

### Quantitative differences in gene expression in *N. benthamiana* after ZNC treatment

**Fig. 5:**
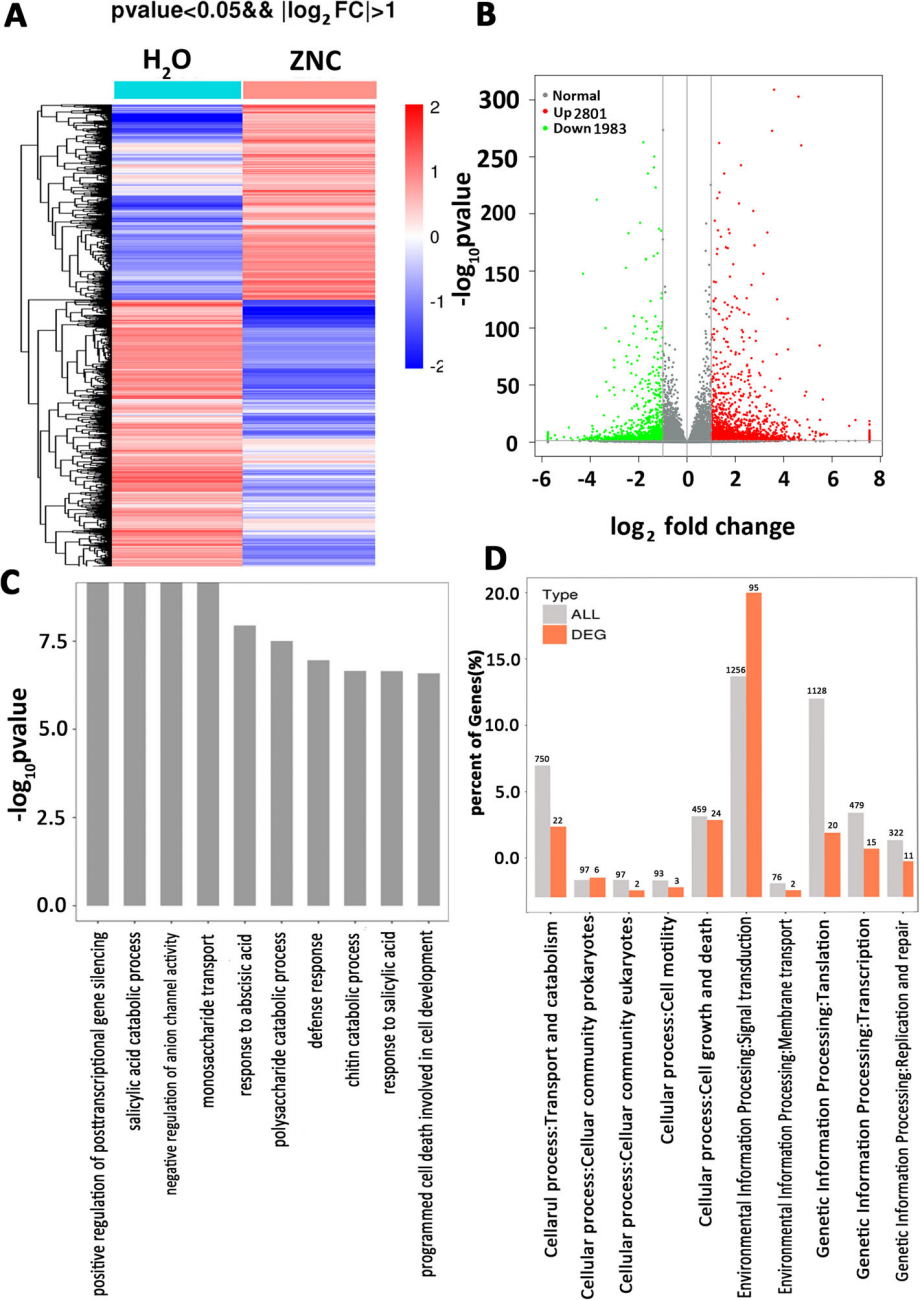
Transcriptome analysis after ZNC treatment in *Nicotiana. Benthamiana*. RNA samples were taken from five or six leaf stage leaves, which were treated with 0 (H_2_O) or 150 ng/mL of ZNC for 2 h. Each treatment contain three samples (plants). **a** A set of comparison heatmap for ZNC treatment at 2 hpi. The red color in the figure indicates the high-expression genes, and the blue color indicates the low-expression genes. **b** Volcano plot showing fold change and adjusted *p*-value of normalized read counts of the transcriptome sequencing data. The criteria of log_2_| (fold change) | ≥ 1 and padj ≤0.05 were used to identify the DEGs. Green dots indicate the downregulated DEGs (1983 genes), and red dots indicate the upregulated DEGs (2801 genes). **c** GO enrichment top 10 (upregulated) term of biological process. The numbers of -log_10_*P* value of differential genes between H_2_O and ZNC groups in every term > 2 were screened, and the top 10 terms were sorted by the -log_10_*P* value corresponding to each term. **d** Partial KEGG pathway classification. Abscissa axis is the ratio of DEG in a pathway: all DEG in KEGG level 2 pathway (%), ordinate axis is the name of pathway. The numbers above the column represent the quantity of DEG in the pathway.

To further reveal the role of ZNC in inducing plant resistance, a nonparametric transcriptome sequencing analysis was performed on leaves treated with ZNC. The leaves were harvested after 2 h of 150 ng/mL ZNC treatment. More than 47 million clean reads were generated (Table S2). A total of 2801 genes were upregulated twofold, and 1983 genes were downregulated (*P* < 0.05) in *N. benthamiana* plants treated with 150 ng/mL of ZNC compared with those treated with H_2_O at 2 hpt (Fig. 5a and b). Most of the ZNC-regulated genes at 2 hpt belong to a very wide range of GO categories, such as those related to biological processes, cellular component, and molecular function (Fig. S4). The upregulated GO term (Table 1) of biological process included positive regulation of PTGS, defense response, and response to SA (Fig. 5c). Within KEGG classification, plant hormone signal transduction genes showed the highest expression changes, and 95 differentially expressed genes (DEG) were upregulated (Fig. 5d and Fig. S5). Thus, we speculate that the resistance to PVX was induced by RNA silencing via SA pathway.

**Table 1:** Gene ontology (GO) enrichments of upregulated genes

GO_Accession	Description (Term)	DEG Number	category
GO:0060148	positive regulation of posttranscriptional gene silencing	3	biological_process
GO:0046244	salicylic acid catabolic process	3	biological_process
GO:0010360	negative regulation of anion channel activity	5	biological_process
GO:0015749	monosaccharide transport	3	biological_process
GO:0009737	response to abscisic acid	40	biological_process
GO:0000272	polysaccharide catabolic process	9	biological_process
GO:0006952	defense response	43	biological_process
GO:0006032	chitin catabolic process	8	biological_process
GO:0009751	response to salicylic acid	18	biological_process
GO:0010623	programmed cell death involved in cell development	4	biological_process
GO:0004568	chitinase activity	8	molecular_function
GO:0010200	response to chitin	12	biological_process

### ZNC enhanced RNA silencing

**Fig. 6:**
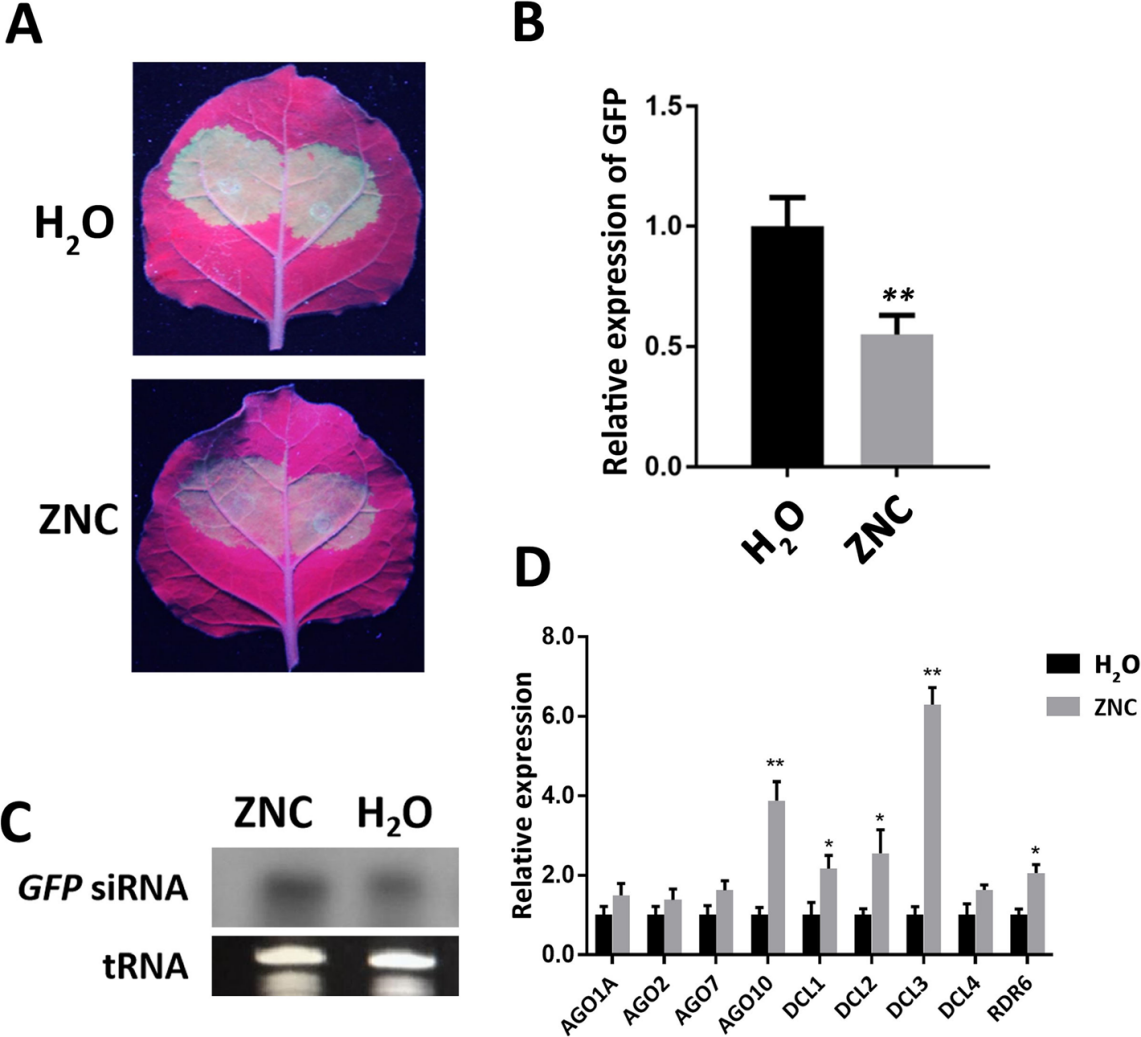
ZNC enhanced RNA silencing. **a***N. benthamiana* leaves were inoculated with *Agrobacterium* harboring *PBI121-GFP* after water (up panel) or 150 ng/mL of ZNC (down panel) treatment for 2 h. Then, the leaves were examined under UV light at 3 dpi. **b** The relative expression level of GFP was detected by qRT-PCR. Data are shown as the mean (*n* = 3) ± SD, and ** indicates extremely significant differences determined using the Student’s t-test (*p* < 0.01). **c** Northern blot analysis of *GFP* siRNA extracted at 3 dpi from patches, the cropped gel and blot images were shown in Fig. 6c, and tRNA was used as loading control for siRNA. **d** The relative expression of genes related to RNA silencing pathway was detected using qRT-PCR after ZNC treatment for 2 h. Data are shown as the mean (*n* = 3) ± SD. * indicates significant differences determined using the Student’s t-test (*p* < 0.05), and ** indicates extremely significant differences determined using the Student’s t-test (*p* < 0.01).

The results of transcriptome sequencing showed that the ZNC could positively regulate the PTGS. Thirty *N. benthamiana* were sprayed with ZNC and deionized water to study the effect of ZNC on RNA silencing. After 2 h, the *Agrobacterium* culture (OD_600_ = 1.0) harboring pBI121-GFP was injected into leaves, and RNA silencing was determined by observing the GFP fluorescence intensity in the infiltrated and newly emerging leaves under long-wavelength ultraviolet light for 7 days. Then, GFP emerging leaves were collected, the total RNA was extracted, and the expression levels of GFP were detected by qRT-PCR. As shown in Fig. 6, the GFP fluorescence intensity was strongest at 3 dpi, and the GFP fluorescence intensity and expression level of the ZNC treatment group were significantly lower than those of the control group at 3 dpi (Fig. 6a and b). Moreover, siRNA was extracted, and Northern blot was performed. The *GFP* siRNA accumulation of the ZNC treatment group was higher than that of the control group (Fig. 6c). The above-mentioned results suggest that ZNC promotes RNA silencing.

DCL and AGO are the key proteins in the RNA-silencing pathway. DCL is responsible for cutting the dsRNA produced by cells, and AGO acts as a catalytic component of RNA-induced silencing complex (RISC), which promotes amplified silencing signal. The genes related to RNA-silencing pathway were detected by qRT-PCR. *NbDCL1*, *NbDCL2*, *NbDCL3*, *NbDCL4*, *NbRDR6*, *NbAGO7*, and *NbAGO10* were upregulated; among these genes, *NbDCL3* and *NbAGO10* were significantly upregulated (Fig. 6d). Hence, ZNC plays a role of silencing enhancement by regulating the upstream pathway of RNA silencing.

### ZNC promoted RNA silencing through SA pathway

**Fig. 7:**
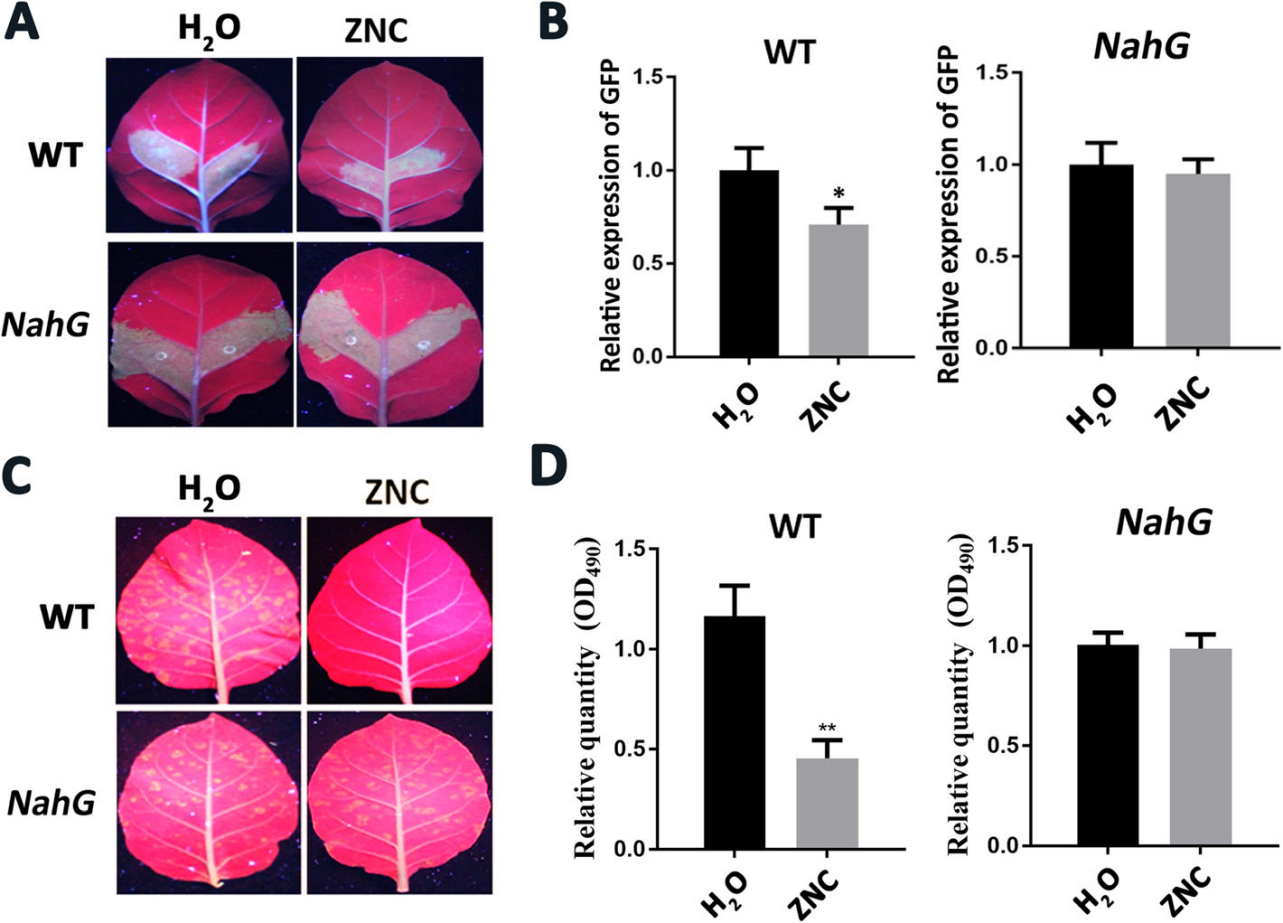
ZNC induced the antiviral activity of plants by increasing RNA silencing via SA pathway. **a** Photograph of wide type *N. tabacum* cv *Samsun NN* (up panel) and *NahG* transgenic *N. tabacum* cv *Samsun NN* (down panel) infiltrated with *Agrobacterium* harbouring *PBI121-GFP* after water (left panel) or ZNC (right panel) treatment, under an ultraviolet lamp at 3 dpi. **b** The relative expression levels of GFP from wild-type plants and *NahG* transgenic plants were detected by qRT-PCR. Data are shown as mean (*n* = 3) ± SD. * indicates significant differences determined using the Student’s t-test (*p* < 0.05). **c** Effect of ZNC on PVX-GFP accumulation in wild-type *N. tabacum* cv *Samsun NN* (up panel) and *NahG* transgenic *N. tabacum* cv *Samsun NN* (down panel). The photographs were taken under an ultraviolet lamp at 3 dpi. The plants were treated with water (left panel) or ZNC (right panel). **d** The relative expression relative quantity of PVX from wild-type plants and *NahG* transgenic plants were detected by qRT-PCR. Data are shown as mean (*n* = 3) ± SD. * *indicates significant differences determined using the Student’s t-test (*N* < 0.01).

Previous studies have shown SA induce RNA silencing-related genes and plant resistance to RNA pathogens [28]. So we speculate that ZNC induced virus resistance by RNA silencing through SA pathway. To test this speculation, *NahG* transgenic *N. tabacum* cv *Samsun NN* was used as plant material. SA could not accumulate because SA was broken down into catechins. In a transient expression experiment, we could see that the fluorescence intensity of GFP was the strongest at 3 dpi emerging area and then gradually decreased. However, the GFP fluorescence intensity showed no significant change between ZNC treatment leaves and water treatment group, and the expression levels of *GFP* from the two treatment areas were consistent with the phenomenon (Fig. 7a and b). On the contrary, the GFP fluorescence intensity of the nontransgenic plant treated with ZNC was significantly decreased compared with that of the nontransgenic plant sprayed with water, and the *GFP* expression level was consistent with the fluorescence intensity.

To further confirm the results, *NahG* transgenic *N. tabacum* cv *Samsun NN* and wild-type *N. tabacum* cv *Samsun NN* plants were inoculated with PVX by rubbing leaves with sap. The PVX accumulation (Fig. 7d) was more and the emerging area was larger in the water treatment group than in the ZNC treatment group in the wild-type tobacco at 15 dpi. For *NahG* transgenic *N. tabacum* cv *Samsun NN*, the PVX accumulation and emerging area showed no significant difference (Fig. 7c). The results indicate that ZNC cannot induce antivirus ability for *NahG* transgenic *N. tabacum* cv *Samsun NN* and SA-deficient plants. All the results mentioned above suggest that ZNC promotes RNA silencing through SA pathway and then induces virus resistance.

## Discussion

Most plants possess endophytes (mostly bacteria and fungi), and endophytes can promote growth and plant health in most cases. During long-term coevolution with plants, endophytic bacteria and fungi have developed many factors that help plants resist stresses and promote their growth. For instance, endophytic microbes are often functional in that they may increase the stress tolerance of plants, enhance disease resistance in of plants, suppress virulence in pathogens, restrain the development of competitor plant species, and carry nutrients from soil into plants. Endophytic microbes may significantly reduce the use of agrochemicals (fertilizers, fungicides, insecticides, and herbicides) in the cultivation of crop plants because of the effective functions of endophytic microbes [44–46]. However, the number of biocontrol agents used in agriculture is very small. ZNC, which is a commercialized product, is the crude extract of a *P. variotii* strain, which is isolated from the roots of plants [9]. ZNC has been used on crops, vegetables and fruit trees widely, and the molecular mechanisms of plant growth promotion and bacterial disease protection have been evaluated recently. To further understand the antiviral mechanism of ZNC, we investigated ZNC-mediated virus resistance in tobacco. In our study, we found that ZNC cannot cure virus-infected plants, but it has prophylactic function, enhances the resistance to virus in plants (Fig. 1 and S3). ZNC can promote H_2_O_2_ (Fig. 2), SA accumulation (Fig. 3), and activate RNA silencing (Fig. 6a and b). These data confirm that ZNC acts as an elicitor of active defense responses in plants. The antiviral mechanism of ZNC was first revealed in this study.

Many elicitors, which can induce plant defense response and protect them from pathogen infection have been identified [47]; they include flagellin [48], elongation factor [49], chitin [50, 51], and other oligosaccharide (e.g., oligogalacturonide acid, OGs) [52]. They are pathogen-associated molecular patterns (PAMPs) and interact with plant receptors that active the PAMP-triggered immunity [53–55]. However, the above-mentioned elicitor’s effective concentration is between μg/mL and mg/mL in order of magnitude, which is considerably higher than the working concentration of ZNC (ng/mL), such as Flg22 (1 μM, approximately 2.232 μg/mL) and OGs (50 μg/mL) [48]. Moreover, several plant hormones’ working concentration is higher than that of ZNC; for example, the antiviral concentration of SA is 10 μM, approximately 1.38 μg/mL [26]. ZNC is the fermentation extract from *p. variotii*, mainly containing nucleoside, saccharides, protein and other compounds (data not shown). We do not know which molecular is an effective elicitor that induces plant resistance to virus because we cannot acquire single compound. Hence, further research is required to separate, extract, and identify effective compounds.

Abiotic stress generally enhances the production of cellular reactive oxygen species (ROS) in plants [56]. Previously studies have shown that SA and hydrogen peroxide levels are closely correlated, and they mutually induce each other’s accumulation during many biological processes [57–59]. Virus invasion causes plant hypersensitivity reaction and generates abundant hydrogen peroxide, which accelerate the cells into programmed cell death, inhibit the reproduction and spread of the virus, and enhance the resistance to virus of plant. So we believe that hydrogen peroxide plays an integral role as signaling molecules in the regulation of numerous biological processes such as responses to abiotic stimuli in plants [60, 61]. Plants have ROS scavenging activity to prevent plants from its toxic effects. SOD and Rbohs are mainly responsible for generating hydrogen peroxide, which can convert harmful superoxide radicals into hydrogen peroxide, and then ROS scavenging enzymes APX and/or CAT hydrolyze hydrogen peroxide. In this study, we found that DAB staining showed a significant difference after ZNC treatment, whereas NBT staining showed no significant difference. *NbCAT* gene related to the hydrogen peroxide-scavenging process was significantly downregulated, whereas *RbohB* gene was upregulated. ZNC may promote the accumulation of hydrogen peroxide by promoting the accumulation of SA; this finding needs further study.

SA biosynthesis induced by pathogen infection mainly depends on the *ICS1* gene and partly depends on *PAL* gene. The ICS1 protein has isochorismate synthase activity, whereas PAL is a phenylalanine ammonia lyase [62–64]. Interestingly, in our study the SA biosynthesis mainly depends on *PAL* gene (Fig. 4a). The reason is unclear and requires further research.

The phytohormone SA plays important roles in regulating disease resistance. Systemic acquired resistance (SAR) depends on SA signaling in plants, such as the chemical benzothiadiazole induces SAR by increasing endogenous SA levels [65, 66]. Previous studies have shown that exogenous SA also increases PR gene expression [67]. Thus, SAR requires signal molecule SA and is associated with the accumulation of PR proteins, which are supposed to contribute to resistance [22]. In our study, the SA content in plants increased after spraying ZNC, and the related marker genes *PR1A*, *NPR1*, *WRKY40*, *WRKY51* and *WRKY 70* in SA pathway were upregulated by qRT-PCR (Fig. 4b). These results confirm that the biosynthesis of SA dependent on PAL is required in the ZNC-mediated defense response.

Transcriptome sequencing showed that many different genes were upregulated; such genes are involved in various aspects, such as positive regulation of PTGS, SA catabolic process, response to SA, and defense response (Fig. 5, Fig. S4, S5). The aforementioned biological process is closely related to plant defense and induction of resistance. Previous studies have shown that SA induces RNA-silencing-related genes [27, 28]. Moreover, the RNA-silencing pathways modulate responses to certain stresses and can be partially tuned by SA [68], and SA-related transcription factors are coexpressed with several AGO and DCL [69]. In our study, we found that NbDCL1, NbDCL2, NbDCL3, NbRDR6, NbAGO7, and NbAGO10 were upregulated with SA accumulation in the marker genes of RNA-silencing pathway (Fig. 6). The results suggest that preinduction of RNA-silencing-related genes by SA inhibits PVX accumulation.

Previous studies showed that SA induces RNA silencing-related genes and plant resistance to virus [28], and SA-deficient *N*. *benthamiana* attenuates virus-induced gene silencing but does not affect transgene-induced PTGS [70]. Our study showed that ZNC could promote the accumulation of SA (Fig. 4), and transcriptome sequencing results showed that ZNC upregulated the expression of RNA silencing-related genes (Fig. 5). Silencing efficiency analysis showed that the fluorescence intensity of wild-type plants treated with ZNC was significantly weaker than that of the water group. However, no significant difference in fluorescence intensity in *NahG* transgenic plants was observed (Fig. 7a and b). Furthermore, ZNC-sprayed wild-type leaves showed decreased infection areas, whereas ZNC failed to induce a protective effect against PVX in *NahG* leaves. These results indicated that virus resistance can be achieved by RNA silencing via the SA pathway for ZNC. However, RNA silencing is dispensable for SA-mediated resistance against plant RNA viruses in *Arabidopsis thaliana* [71]. These findings suggest that SA may increase plant resistance to viruses in various of parallel resistance mechanisms, such as inducing systemic acquired resistance (SAS) [72] or acting as an enhancer of the RNA silencing antiviral defense in tobacco. SA and RNA silencing defense mechanisms may also cooperate to prevent virus invasion [73–75]. Thus, we presumed that ZNC increases plant viral resistance by enhancing RNA silencing via the SA pathway, which is a mechanism to fight diseases. Whether ZNC can improve resistance to the virus through other SA pathways remains unclear.

Furthermore, SA was found in numerous plants (*Nicotiana tabacum*, *tomato*, *Arabidopsis thaliana*, *Orange*), and could induce RNA silencing-related genes and plant resistance to RNA pathogens [28, 75]. The previous report showed that ZNC activated the salicylic acid biosynthesis and signaling pathways in *Arabidopsis thaliana* [9]. In the support information, we found that ZNC also enhance N.*benthamiana* resistance to TMV. Therefore, we speculated that ZNC can induce the resistance to various virus through SA and RNA silencing pathway in different plants. The antiviral phenomenon of ZNC is nonspecific.

## Conclusions

We firstly demonstrated that ultrahigh-activity ZNC could induce virus resistance at low concentration, and the antiviral mechanism was preliminary revealed. All results indicated that ZNC mediated resistance to viruses by positively regulating RNA silencing via salicylic acid SA pathway. This study provides a new antiviral bioagent, namely, ZNC. This bioagent will be very useful bioagent for plant antiviral research and application.

## Methods

### Materials and growth conditions

ZNC was provided by Shandong Pengbo Biotechnology Co., Ltd.; it is an ethanol crude extract of *Paecilomyces variotii*. The pCaPVX440-GFP-YFP plasmids were presented by professor Xiangdong Li (Shandong agricultural university, china) [76] (Fig. S1). For RNA-silencing analysis, the empty vector pBI121 with GFP tag was transformed into *Agrobacterium tumefaciens* GV3101 in this study. Wild-type *Nicotiana benthamiana* and *N. tabacum* cv *Samsun NN* were propagated and stored in this study. *NahG* (which encodes a salicylate hydroxylase converting SA into catechol) transgenic *N. tabacum* cv *Samsun NN* (from Dr. Josefa M. Allimino, campus universidad atonoma de madrid, Spain) [74] were raised in a greenhouse at 24 °C under a 16-h light/8-h dark photoperiod. The DNA polymerase, and other genetic engineering enzymes were purchased from Takara Inc. (Dalian, China).

All other chemicals and reagents were of the highest quality available.

### Antivirus study of ZNC

The wild-type *N. benthamiana* was inoculated at five or six leaf stage with *Potato Virus X* (PVX)-GFP-YFP before or after spraying 100 ng/mL of ZNC to confirm the resistance of ZNC to the infection of PVX. For virus inoculation, 1 g of PVX-infected *N. tabacum* leaves was ground in 1 mL of 5 mM phosphate buffer with pH 7.2. Control plants at six-leaf stage were inoculated by rubbing leaves with freshly prepared sap. Inoculated plants were grown in an insect-free greenhouse at 24 °C, and the viral symptom was monitored. Each experiment was replicated three times and included 10 independent plants.

A gradient concentration aqueous solution of ZNC at 0, 50, 100, 150, and 200 ng/mL was sprayed on *N. benthamiana* for 2 h before inoculating PVX to further determine the optimal working concentration for the antivirus of ZNC; all other conditions were the same as above. The infection of PVX was determined by observing GFP-YFP fluorescence under long-wavelength (365 nm) UV light (Spectroline Model SB-100P/A; Spectronics Corporation, Lexington, KY, USA) and photographed with a Fujifilm FinePix S8000fd digital camera (Fujifilm Holdings Corporation) [77] . The virus content was detected by enzyme-linked immunosorbent assay (ELISA) [78, 79]. The plates were followed by colorimetric readout using an EnSpire Multilabel Reader at 490 nm wavelength. Control groups were *N. benthamiana* leaves without inoculating PVX.

### Histochemical staining of reactive oxygen species

The *N. benthamiana* leaves of ZNC and H_2_O treatment groups were detached for histochemical staining procedure. Staining was performed using 3,3′-diaminobenzidine (DAB) and nitroblue tetrazolium (NBT) to detect the accumulation of H_2_O_2_ and O_2_^−^. The accumulation of H_2_O_2_ in leaves was visualized histochemically using DAB as an indicator. The leaves were placed into 1 mg/mL of DAB solution, vacuum-infiltrated for 30 min, washed thrice with deionized water and reacted with H_2_O_2_ for 12 h under light at 28 °C. After the reaction, a deep brown deposition was clearly visible in leaves, excess dye solution was washed away with boiled ethanol and leaves were imaged.

For NBT staining, *N. benthamiana* leaves were placed into a 1% (M/V) sodium azide solution, vacuum-infiltrated for 30 min, transferred into a 0.5 mg/mL of NBT solution, followed by another vacuum-infiltration for 30 min. Sodium azide may improve cell permeability for allowing the NBT solution to spread throughout the entire seedling. NBT reacted with O^2−^ and thus formed a dark blue insoluble complex substance. Redundant dye solution was washed away with boiled ethanol, and the leaves were imaged. ImageJ software was used for quantitative analysis of H_2_O_2_ and O_2_^−^intensity [80].

### SA extraction and content analysis

After *N. benthamiana* were sprayed with 150 ng /mL ZNC, the free SA was extracted at different time points (0, 2, 4, 6, 8 h) using the previous methods. Briefly, 1 mL of 70-% methanol was added in a mortar after 0.1 g of seeding samples was grinded and then left overnight at 4 °C. The samples were transferred into polypropylene centrifuge tubes. Extract was obtained by centrifugation (8000×g, 10 min). The supernatant was then placed in another tube for evaporation (N_2_, 45 °C). The dry residues were reconstituted with 500 μL ddH_2_O. A total of 300 μL of extract was reconstituted with 40 μL 0.5 mg/mL Trifluoroacetic Acid (TFA), Mix and shake for 1 min. 1 ml of ethyl acetate and cyclohexane was added for removing organic phase, the aqueous phase was then placed in another tube for evaporation (N_2_, 45 °C). The dry residues were reconstituted with 500 μL mobile phase. Finally, an aliquot of 500 μL of extracting solution was transferred to an EP tube after 0.22 μm filtration (0), [81]. SA extracting solution (10 μL) and SA standard were analyzed using HPLC by monitoring the absorbance at 306 nm. All the samples were loaded on the C18 reverse-phase chromatographic column (250 mm × 4.6 mm, 5 μm), respectively. Separation was eluted methanol and 0.1% acetic acid water (the ratio is 65:35) and then eluted at 35 °C over 30 min with a flow rate of 0.8 mL/min.

### RNA extraction and quantitative real-time PCR

Total RNAs were extracted from leaves using Trizol reagent (TaKaRa, Shiga, Japan) in accordance with the instructions of the manufacturer and treated with DNase I at 37 °C for 30 min prior to reverse transcription. cDNA was synthesized from 1 μg of total RNA using FastKing gDNA Dispelling RT SuperMix kit (Tiangen, Beijing, China). Quantitative real-time (qRT-PCR) was performed using Talent SYBR Green Kit (Tiangen, Beijing, China). Each reaction was conducted in triplicate and repeated three times. The results were analyzed by Bio-Rad CFX Manager software (Bio-Rad, California, USA). The relative expression of SOD, CAT, APX, *RbohA*, and *RbohB* were measured using qRT-PCR. Each assay was repeated three times. Primer sequences for the qRT-PCR experiment are presented in Table S1.

### Data analysis of RNA-sequencing

RNA samples were taken from five or six leaf stage leaves, which were treated with 0 (H_2_O) or 150 ng/mL of ZNC for 2 h. Each treatment contain three samples (plants). The samples were selected depending on quality (RIN score ≥ 7). They were pooled and sequenced by Shanghai OE Biotech. Co., Ltd. (Shanghai, China). Previous articles have provided a detailed description of how to analyze RNA-sequening data [82, 83]. All differential gene expressions were based on the following standard: the absolute value of log2 ratio ≥ 1 and FDR ≤ 0.001.

### RNA-silencing analysis

The wild-type *N. benthamiana*, *N. tabacum* cv *Samsun NN*, and *NahG* Transgenic *N. tabacum* cv *Samsun NN* leaves were infiltrated at five- or six-leaf stage with Agrobacterium GV3101 carrying the GFP gene. Each Agrobacterium culture (OD_600_ = 1.0) was incubated for 3 h and then mixed with other culture (s) in a 1:1 (v/v) ratio prior to infiltration. Local and systemic RNA silencing were determined by observing GFP-YFP fluorescence in the infiltrated and newly emerging leaves under long-wavelength (365 nm) UV light as described above. Silencing pathway-related genes, such as (AGO) protein, DCL protein, and RDRP were also detected by qTR-PCR.

### Northern blot analysis of siRNA

Low-molecular-weight RNAs were extracted from leaves as described previously [84]. For siRNA detection, 15 μg of Low-molecular-weight RNAs was separated on 15% polyacrylamide–7 M urea gel and transferred to a Hybond-N^+^ membrane in 0.5 X TBE at 0.8 mA cm^− 2^ for 1 h. After the membrane was UV-crosslinked and incubated at 80 °C for 2 h, it was hybridized with DIG labeled probe GFP mRNA. Chemiluminescent detection was conducted using a DIG Northern Starter Kit (Roche, Basel, Switzerland) in accordance with the instructions of the manufacturer.

## Supplementary information

**Additional file 1: Fig. S1.** Genomic structure of vector pCaPVX440-GFP-YFP. The gfp gene was cloned into pCaPVX440 in the *Asi*SI site, while yfp gene was inserted between the *Sac*I and *Mlu*I site. RdRp, RNA-dependent RNA polymerase; 25 K, 12 K and 8 K are movement protein; CP, coat protein.

**Additional file 2: Fig. S2.** The effection of ZNC when give prior to PVX. Wild-type *N. benthamiana* was inoculated with PVX (GFP + YFP tag) before ZNC (150 ng/mL) treatment for 2 h, the phenotype (**A**) was obtained under long-wave ultraviolet lamp, and PVX relative expression quantity (**B**) was calculated using ELISA method at 5 dpi. Error bars show the mean ± SD of three replicates (at least 20 plants per replicate). ** indicates extremely significant differences determined using the Student’s t-test (*p* < 0.01).

**Additional file 3: Fig. S3.** ZNC treatment enhanced plant resistance against TMV. Wild-type *N. benthamiana* was inoculated with TMV (GFP tag) after ZNC (100 ng/mL) treatment for 2 h. The phenotype was obtained under irradiation with long-wave ultraviolet lamp at 7 dpi. (A) Inoculated leaves. (B) Whole plants.

**Additional file 4: Fig. S4.** Top 30 GO enrichment terms. Differential genes between H_2_O and ZNC groups with a -log_10_*P* value of greater than 2 in every term were screened, and the top 10 terms were sorted by the -log_10_*P* value corresponding to each term. Case: 150 ng/mL ZNC; control: 0 ng/mL ZNC.

**Additional file 5: Fig. S5.** Kyoto Encyclopedia of Genes and Genomes (KEGG) enrichment analysis of differentially expressed genes (DEGs) in the plant in response to ZNC. The top 20 enriched pathways in the plant. Each circle represents a KEGG pathway, the Y-axis represents the pathway name, and the X-axis represents the enrichment score, which compares the ratio of genes annotated to a pathway among the DEGs to the ratio of genes annotated to that pathway among all genes. The larger the enrichment factor, the more significant the enrichment of DEGs in the pathway.

**Additional file 6: Fig. S6.***GFP* siRNA accumulation. Northern blot analysis of *GFP* siRNA extracted at 3 dpi from patches, tRNAs stained by ethidium bromide were shown as loading controls for siRNAs. The pictures in the box are the same as Fig. 6c.

**Additional file 7: Table S1** Primers used for sequencing in the present study. **Table S2** The number of reads obtained from each sample. ZNC/H_2_O: the treatment with ZNC or distilled water.

## Data Availability

The Transcriptome Sequence data are deposited the in the NCBI Sequence Read Archive (SRA) under accession number PRJNA616072. The datasets analyzed during the current study are available from the corresponding author on reasonable request. All data generated or analyzed during this study are included in this published article [and its supplementary information files].

## Abbreviations

DAB: 3,3-diaminobenzidine
H_2_O_2_: Hydrogen peroxide
NBT: Nitroblue tetrazolium
*NPR*: Nonexpressor of pathogenesis-related
PAL: Phenylalanine ammonia lyase
PR: Pathogenesis-related protein
PVX: *Potato virus X*
ROS: Reactive oxygen species
SA: Salicylic acid
TMV: *Tobacco mosaic virus*
ZNC: ZhiNengCong
