# Supplementary figures and images for: Ultrahigh-activity immune inducer from Endophytic Fungi induces tobacco resistance to virus by SA pathway and RNA silencing
Source: BMC Plant Biol. 2020 Apr 15;20:169. doi: 10.1186/s12870-020-02386-4 (PMC7160901; doi:10.1186/s12870-020-02386-4)

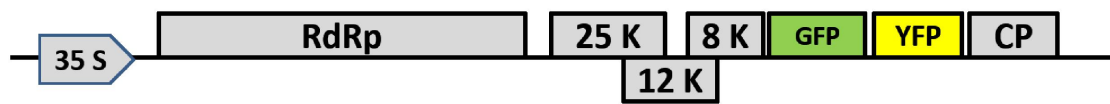

Supplement: Supplementary file 1 — Additional file 1: Fig. S1. Genomic structure of vector pCaPVX440-GFP-YFP. The gfp gene was cloned into pCaPVX440 in the AsiSI site, while yfp gene was inserted between the SacI and MluI site. RdRp, RNA-dependent RNA polymerase; 25 K, 12 K and 8 K are movement protein; CP, coat protein. [file 12870_2020_2386_MOESM1_ESM.pdf]

**A**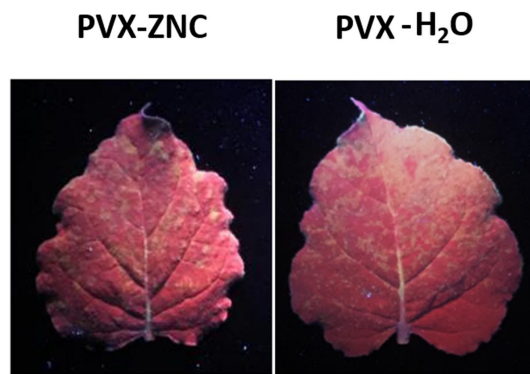**B**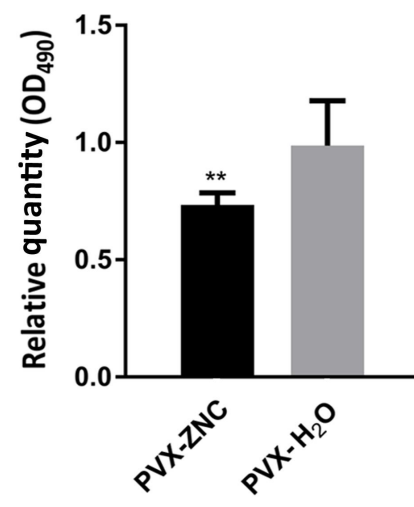

Supplement: Supplementary file 2 — Additional file 2: Fig. S2. The effection of ZNC when give prior to PVX. Wild-type N. benthamiana was inoculated with PVX (GFP + YFP tag) before ZNC (150 ng/mL) treatment for 2 h, the phenotype (A) was obtained under long-wave ultraviolet lamp, and PVX relative expression quantity (B) was calculated using ELISA method at 5 dpi. Error bars show the mean ± SD of three replicates (at least 20 plants per replicate). ** indicates extremely significant differences determined using the Student’s t-test (p < 0.01). [file 12870_2020_2386_MOESM2_ESM.pdf]

**A**

**ZNC**

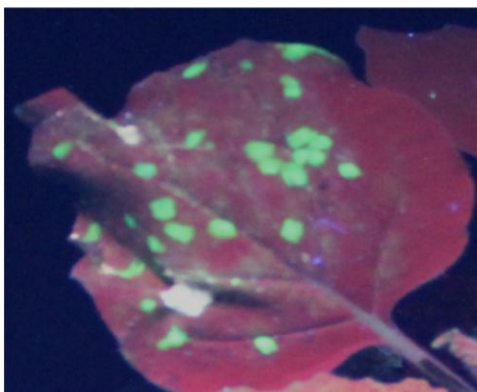

**H<sub>2</sub>O**

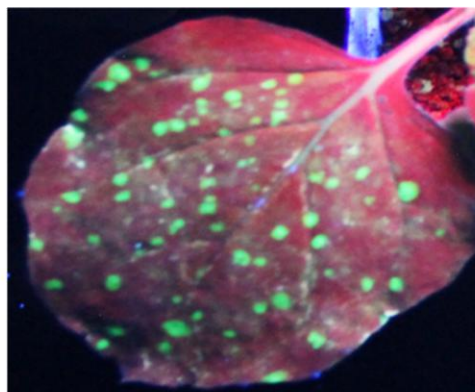

**B**

**ZNC**

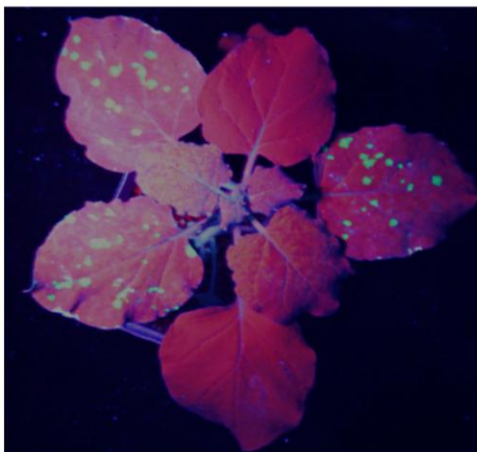

**H<sub>2</sub>O**

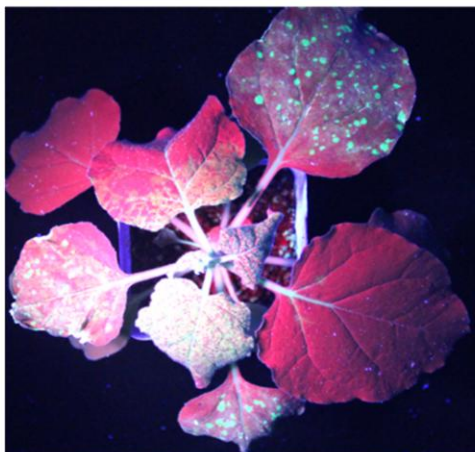

Supplement: Supplementary file 3 — Additional file 3: Fig. S3. ZNC treatment enhanced plant resistance against TMV. Wild-type N. benthamiana was inoculated with TMV (GFP tag) after ZNC (100 ng/mL) treatment for 2 h. The phenotype was obtained under irradiation with long-wave ultraviolet lamp at 7 dpi. (A) Inoculated leaves. (B) Whole plants. [file 12870_2020_2386_MOESM3_ESM.pdf]

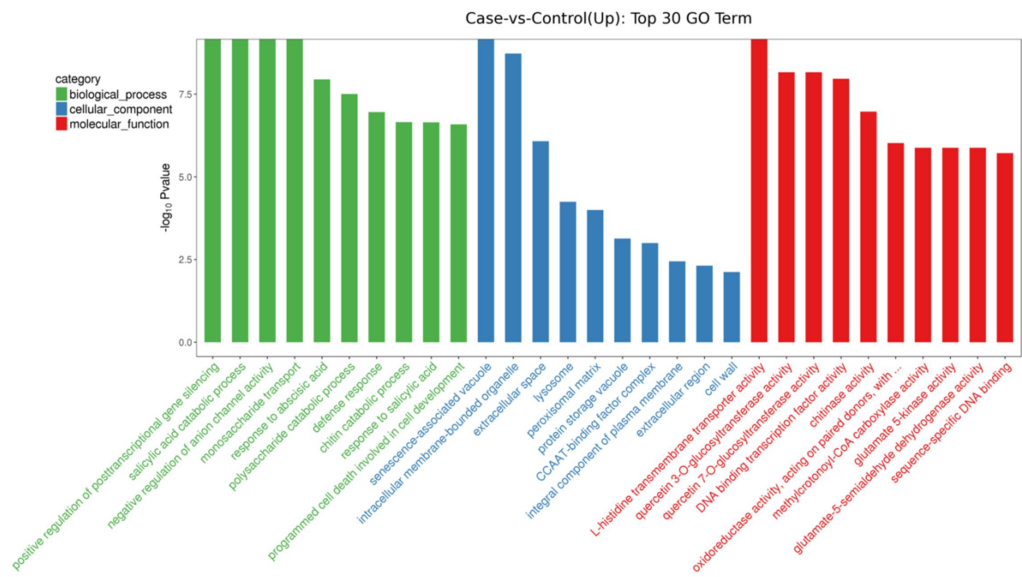

Supplement: Supplementary file 4 — Additional file 4: Fig. S4. Top 30 GO enrichment terms. Differential genes between H2O and ZNC groups with a -log10P value of greater than 2 in every term were screened, and the top 10 terms were sorted by the -log10P value corresponding to each term. Case: 150 ng/mL ZNC; control: 0 ng/mL ZNC. [file 12870_2020_2386_MOESM4_ESM.pdf]

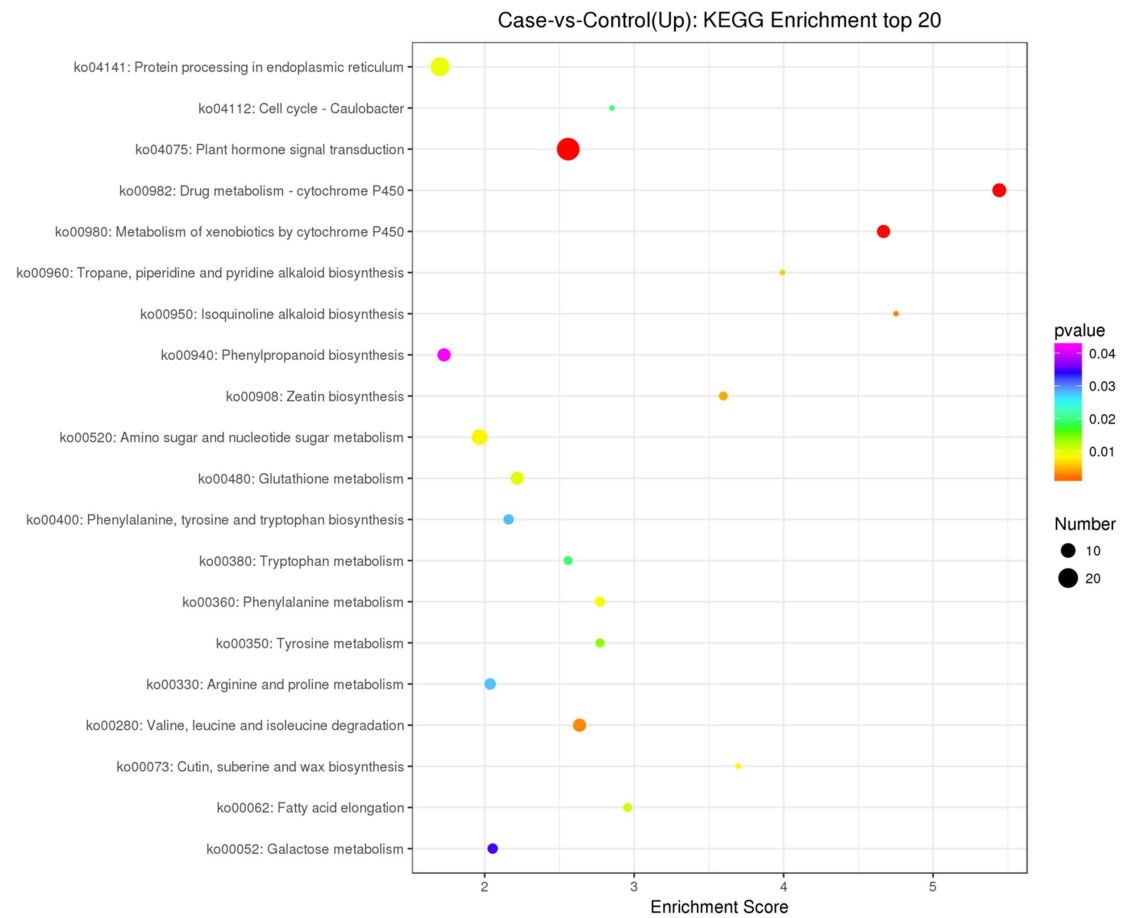

Supplement: Supplementary file 5 — Additional file 5: Fig. S5. Kyoto Encyclopedia of Genes and Genomes (KEGG) enrichment analysis of differentially expressed genes (DEGs) in the plant in response to ZNC. The top 20 enriched pathways in the plant. Each circle represents a KEGG pathway, the Y-axis represents the pathway name, and the X-axis represents the enrichment score, which compares the ratio of genes annotated to a pathway among the DEGs to the ratio of genes annotated to that pathway among all genes. The larger the enrichment factor, the more significant the enrichment of DEGs in the pathway. [file 12870_2020_2386_MOESM5_ESM.pdf]

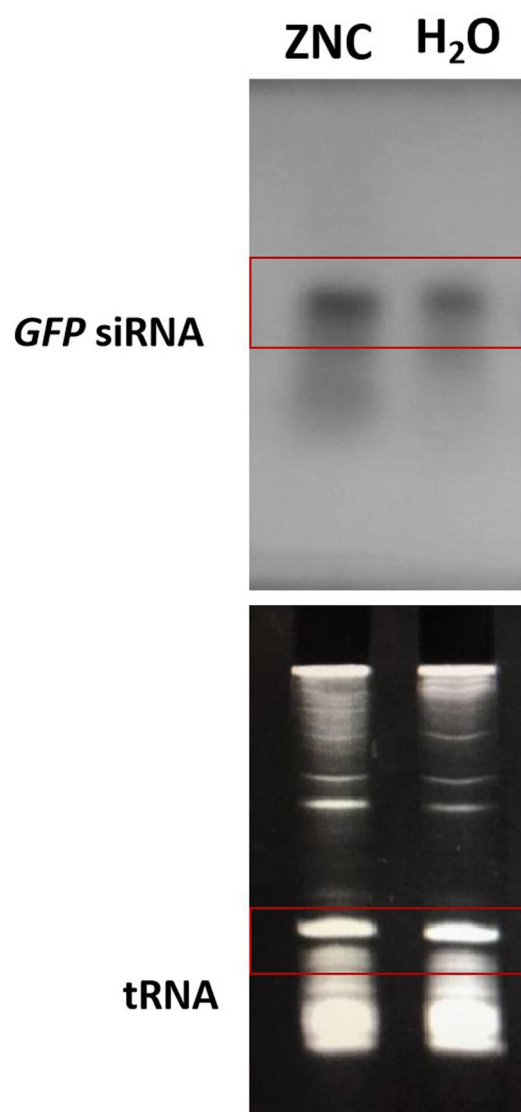

Supplement: Supplementary file 6 — Additional file 6: Fig. S6.GFP siRNA accumulation. Northern blot analysis of GFP siRNA extracted at 3 dpi from patches, tRNAs stained by ethidium bromide were shown as loading controls for siRNAs. The pictures in the box are the same as Fig. 6c. [file 12870_2020_2386_MOESM6_ESM.pdf]
