# Supplementary material for: Ultrahigh-activity immune inducer from Endophytic Fungi induces tobacco resistance to virus by SA pathway and RNA silencing
Source: BMC Plant Biol. 2020 Apr 15;20:169. doi: 10.1186/s12870-020-02386-4 (PMC7160901; doi:10.1186/s12870-020-02386-4)
Supplement: Supplementary file 7 — Additional file 7: Table S1 Primers used for sequencing in the present study. Table S2 The number of reads obtained from each sample. ZNC/H2O: the treatment with ZNC or distilled water. [file 12870_2020_2386_MOESM7_ESM.pdf]

Table S1 primers used for sequencing in the present study.

**Sequencing primers**	Primer sequence (5 '-3')
Nb actin F	TTGGCTTACATTGCTCTTG
Nb actin R	TCATTGATGGTTGGAACAG
qRT-*NbCAT* F	CACTCACCTTACCTGTGCTG
qRT-*NbCAT* R	GAACTTCATTCCATCACGG
qRT-*NbAPX* F	CATCAGGCTATTGGAACCC
qRT-*NbAPX* R	GCTCTGTCTTGTCCTCTCTACC
qRT-*NbSOD* F	GCAGCAGTGAAGGTGTTAGC
qRT-*NbSOD* R	GGATTGTAATGTGGTCCCG
qRT-*NbRbohA* F	CATAACTACTTGACGAGTG
qRT-*NbRbohA* R	CTGGTGCCTGATACAATA
qRT-*NbRbohB* F	CACTATGCTTCAGTCTCTTC
qRT-*NbRbohB* R	GTCTTAGTTCTTTGGTCAG
qRT-*NbPAL* F	CTCAAGTTGCGGCTATTG
qRT-*NbPAL* R	CATTCTTGGTCCTTCTATGTG
qRT-*NbICS* F	CAGTTGAAGAGCAGATAGAAG
qRT-*NbICS* R	AAGTTCCATTGAAGCACATT
qRT-*NbPR-1*A F	CAGCTTGATGTATGATCTGATAT
qRT-*NbPR-1*A W	CTGGTAGATTATATGAACTGTGTAA
qRT-*NbWRKY40* F	TTAGTCCTAAAGGTGGCAATA
qRT-*NbWRKY40* W	TGTCTGATTATTATGTTGGAGATT
qRT-*NbWRKY51* F	AAGAAAGAGAAGATGAATGAAAGA
qRT-*NbWRKY51* W	GCACTTGTAGTAATTCCTTAGAT
qRT-*NbWRKY70* F	ATTCTACCAATAATCATCACCATT
qRT-*NbWRKY70* W	CACCTATCCAGAACCACATA
qRT-*NbRDR6* F	AACTGCTGATGATGATATTGAA
qRT-*NbRDR6* R	CTCCACTACCTGTAATGTCT

qRT-*NbAgo1a* F	ATGAGAGGCAGATAACAG
qRT-*NbAgo1a* R	CAGCATAAGCATTGTGAT
qRT-*NbAgo6* F	CTGCTTCTGGACTTCTATA
qRT-*NbAgo6* R	ACTTGACTGAACTGTGAT
qRT-*NbAgo7* F	TGCTGTAGTTGGTAATGTG
qRT-*NbAgo7* R	ATCTCCTGCCTATGTGTT
qRT-*NbAgo10* F	AGCAAGCAAGCCAGTTAT
qRT-*NbAgo10* R	GAACCAGCAGCCACTAAT
qRT-*NbDCL1* F	ACTCCAATCATACATCCA
qRT-*NbDCL1* R	GTCATACACAGGTAACAC
qRT-*NbDCL3* F	TTATGCTGCTGTGAAGAG
qRT-*NbDCL3* R	CAAGAATCAGAGACCAACT
qRT-*NbDCL4* F	TATTCATTCGTCCATAAG
qRT-*NbDCL4* R	ACACAGAGTATAGATAGG

Table S2 The number of reads obtained from each sample. ZNC / H_2O: the treatment with ZNC or distilled water.

Samples	Raw_reads	Clean_reads
ZNC_1	49705072	6918284150
ZNC_2	49715752	6928642160
ZNC_3	49138846	6845500008
H_2O_1	49091720	6815317964
H_2O_2	49347032	6865097565
H_2O_3	49673408	6906031776
